# Supplementary material for: Infant diarrheal disease in rhesus macaques impedes microbiome maturation and is linked to uncultured Campylobacter species
Source: Commun Biol. 2024 Jan 5;7:37. doi: 10.1038/s42003-023-05695-0 (PMC10770169; doi:10.1038/s42003-023-05695-0)
Supplement: Supplementary file 2 — Supplementary Information [file 42003_2023_5695_MOESM2_ESM.docx]

**Figure S1: Development of the infant mycobiome and impact of diarrhea.** (**A**) correlation between relative abundance and colony forming units equivalent measured by qPCR. (**B**) Stacked bar plot of fungal ITS taxonomy organized by time-point. Each vertical bar represents a single sample. (**C**) Principal coordinate analysis (PCoA) of fecal mycobiome weighted UniFrac distance (ITS amplicon) colored by host status and timepoint. (**D**) Violin plot of observed amplicon sequencing variants at each time point. (**E**) Differentially abundant taxa between healthy and sick samples at 6 and 12 months (LEfSe, Log_10_ LDA score > 2). DX = Diarrhea.


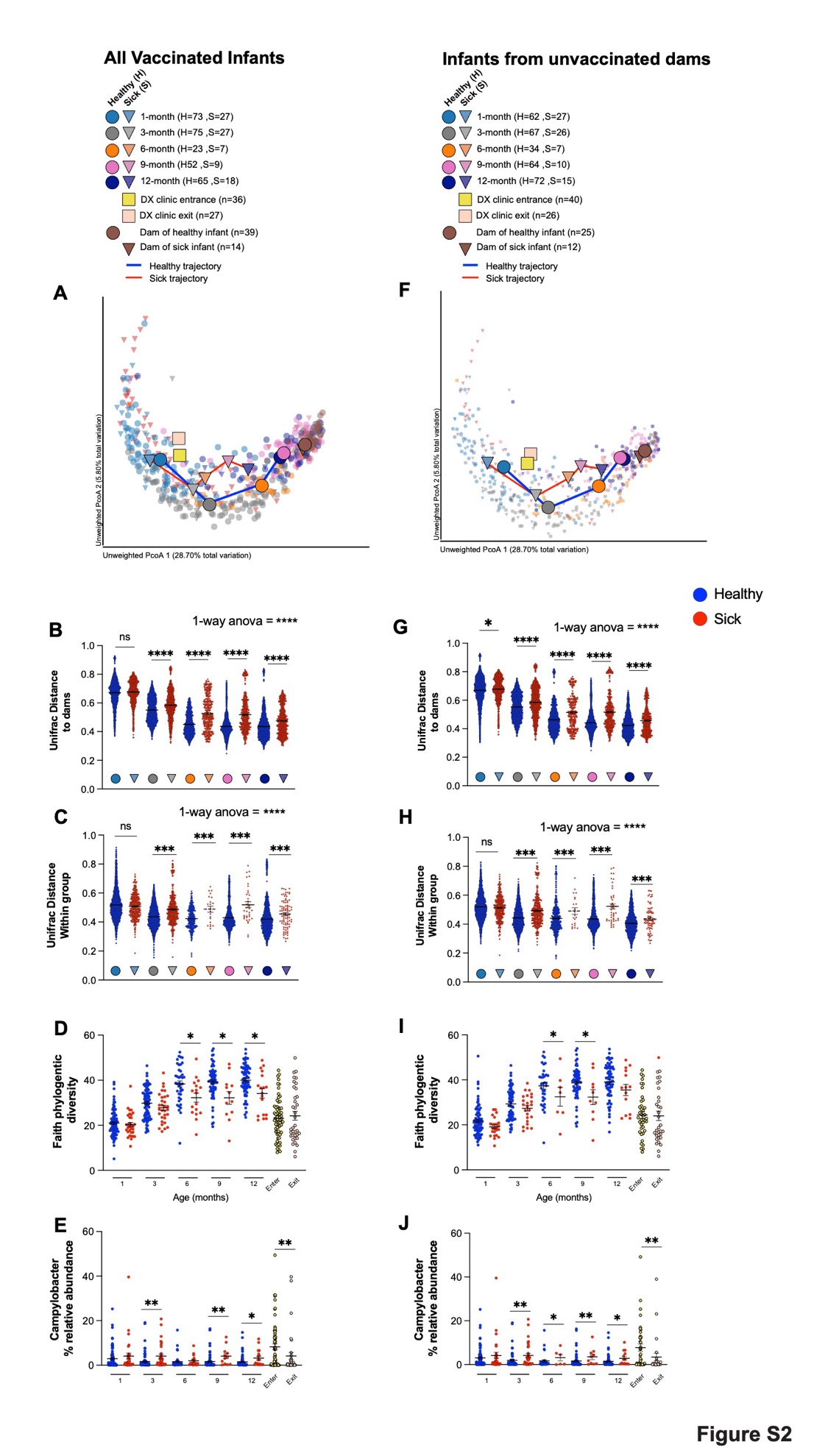


**Figure S2: Vaccination against *C. coli* does not impact microbiome maturation or mitigate impact of breakthrough diarrhea.** Panels **A-E** show data from infants that received a *C. Coli* vaccine to show that vaccination alone did not drive the overall trends observed. Panels **F-J** show data from all infants, both vaccinated and unvaccinated, whose dams were not vaccinated, to illustrate that maternal vaccination had no impact on the observed trends. (**A**) Principal coordinate analysis (PCoA) of fecal microbiome unweighted UniFrac distance (16S amplicon), colored by host status and timepoint. Small points represent individual samples while larger points represent the centroid for a given timepoint/host status. Solid lines connecting centroids illustrate the developmental trajectory of both healthy and sick infants. (**B-E**) Dot plots of **B)** unifrac distance between dams and each infant timepoint/health status, **C)** within group unifrac distance for each infant group, **D)** longitudinal measurements of Faith’s phylogenetic diversity, in healthy and sick infants, and **E)** longitudinal measurements of *Campylobacter* relative abundance, in healthy and sick infants. **F**) Principal coordinate analysis (PcoA) of fecal microbiome unweighted UniFrac distance (16S amplicon), colored by host status and timepoint. Small points represent individual samples while larger points represent the centroid for a given timepoint/host status. Solid lines connecting centroids illustrate the developmental trajectory of both healthy and sick infants. (**G-J**) Dot plots of unifrac distance between dams and each infant timepoint/health status, **H)** within group unifrac distance for each infant group, **I)** longitudinal measurements of Faith’s phylogenetic diversity, in healthy and sick infants, and **J**) longitudinal measurements of *Campylobacter* relative abundance, in healthy and sick infants. Significance for B, C, G, and H was determined using 1-way ANOVA **** p < 0.0001, with Holm-Sidak’s multiple comparison test, **** p < 0.0001, error bars = SEM. Significance for D, E, I and J was determined using unpaired T-test at each time-point, *p <0.05, **p < 0.01, ***p < 0.001, error bars = SEM. DX = Diarrhea.

**Figure S3: The maternal microbiome as a predictor of infant diarrhea.** (**A**) Principal coordinate analysis (PCoA) of fecal microbiome weighted UniFrac dissimilarity (16S amplicon) from maternal samples collected at infant age 1-month, colored by infant status. (**B**) Differentially abundant taxa (16S amplicon) between mothers of healthy and sick infants (LEfSe, Log_10_ LDA score > 2). (**C**) PcoA of Bray-Curtis dissimilarity built on the species level taxonomy generated using MetaPhlAn and colored by infant status. (**D**) Differentially abundant species (MetaPhlAn) between mothers of healthy and sick infants (LEfSe, Log_10_ LDA score > 2). (**E**) PCoA of Bray-Curtis dissimilarity built on the abundance of all functional genes annotated using HUMAnN3 and the Uniref90 database colored colored by infant status. (**F**) Differentially abundant MetaCyc pathways between mothers of healthy and sick infants (LEfSe, Log_10_ LDA score > 2).

**Figure S4: Functional and taxonomic development of the healthy infant microbiome.** (**A**) PCoA of Bray-Curtis dissimilarity built on the abundance of all functional genes annotated using HUMAnN3 and the Uniref90 database colored by and timepoint including only healthy infants and dams. (**B**) PCoA of Bray-Curtis dissimilarity built on the species level taxonomy generated using MetaPhlAn and colored by timepoint including only healthy infants. (**C**) Differentially abundant MetaCyc pathway between time-points including only healthy animals (LEfSe, Log_10_ LDA score > 2). (**D**) Longitudinal average plot of key bacterial species across time-points in healthy infants.

­

**Figure S5:** **UMG-3 lacks motility genes and adhesion/invasion genes are conserved within but not between species.** (**A**) Prevalence heatmap of genes associated with flagellar assembly, chemotaxis, and host adhesion. (**B**, **C**) Phylogeny built on **B)** the amino acid alignment of the adhesion gene cadF and **C)** the *Campylobacter* invasion antigen gene ciaB.
