# Supplementary material for: Infant diarrheal disease in rhesus macaques impedes microbiome maturation and is linked to uncultured Campylobacter species
Source: Commun Biol. 2024 Jan 5;7:37. doi: 10.1038/s42003-023-05695-0 (PMC10770169; doi:10.1038/s42003-023-05695-0)
Supplement: Supplementary file 3 — Description of Additional Supplementary Files [file 42003_2023_5695_MOESM3_ESM.docx]

# Description of Additional Supplementary Data

File Name: Supplementary Tables

Description: All supplemental tables referenced in manuscript

File Name: Supplementary Data 1

Description: The source data behind the graphs in the paper
